# Racially equitable diagnosis of cystic fibrosis using next-generation DNA sequencing: a case report

**DOI:** 10.1186/s12887-021-02609-z

**Published:** 2021-03-31

**Authors:** Bennett O. V. Shum, Glenn Bennett, Akash Navilebasappa, R. Kishore Kumar

**Affiliations:** 1 Preventive Health Division, Genepath, 302B 7 Help St, Chatswood, NSW Australia; 2 EMBL Australia Node in Single Molecule Science, School of Medical Sciences, University of New South Wales, Sydney, NSW Australia; 3 Acquity Labs, 515, 4th Block HBR layout, Bengaluru, Karnataka India; 4 Cloudnine Hospitals, 1533, 3rd Block, Jayanagar, Bengaluru, Karnataka India

**Keywords:** Cystic fibrosis, Genetics, Newborn screening

## Abstract

**Background:**

Cystic Fibrosis (CF) is one of the most prevalent autosomal recessive inherited disease in Caucasians. Rates of CF were thought to be negligible in non-Caucasians but growing epidemiological evidence shows CF is more common in Indian, African, Hispanic, Asian, and other ethnic groups than previously thought. Almost all second-tier molecular diagnostic tools currently used to confirm the diagnosis of CF consist of panels of the most common CF-causing DNA variants in Caucasians. However non-Caucasian individuals with CF often have a different spectrum of pathogenic variants than Caucasians, limiting the clinical utility of existing molecular diagnostic panels in this group. As a consequence of racially inequitable CF testing frameworks, non-Caucasians with CF encounter greater delays in diagnosis and associated harms than Caucasians. An unbiased approach of detecting CF-causing DNA variants using full gene sequencing could potentially address racial inequality in current CF testing.

**Case presentation:**

We present the case of a female baby from rural India who had a borderline first-tier newborn screening result for CF. Instead of choosing a targeted CF panel for second-tier testing, we used next-generation DNA sequencing to comprehensively analyze the cystic fibrosis transmembrane conductance regulator gene as an unbiased approach for molecular confirmation of CF. Sequencing identified two pathogenic variants that cause CF. One variant (c.1521_1523delCTT) is the most common cause of CF, while the other variant (c.870-1G > C) is absent from all population allele databases and has not been found in the Indian population previously. The rare variant would not have been detected by all currently available targeted CF panels used for second- or third-tier molecular CF testing.

**Conclusions:**

Our use of full gene sequencing as a second-tier CF test in a non-Caucasian patient avoided the problems of missed diagnosis from using Caucasian-biased targeted CF panels currently recommended for second-tier testing. Full gene sequencing should be considered as the standard methodology of second-tier CF testing to enable equal opportunity for CF diagnosis in non-Caucasians.

## Background

Cystic Fibrosis (CF) is a multisystem disease that affects the lungs, gastrointestinal tract, pancreas, and other organs, with the major cause of mortality and morbidity resulting from progressive lung disease and chronic respiratory infections [1]. CF is one of the most prevalent autosomal recessive inherited conditions in the Caucasian population, with a birth prevalence of approximately 1 in 2500 [2]. Once thought to be largely restricted to Caucasians, there is growing awareness that CF is more prevalent than previously thought in other ethnic groups, such as Indian, Asian, African, and Hispanic populations [3, 4]. CF is caused by dysfunctional cystic fibrosis transmembrane conductance regulator (CFTR) protein due to DNA variants in the *CFTR* gene, with over 350 disease-causing variations currently described in the widely referenced Clinical and Functional Translation of CFTR project (CFTR2) database (https://cftr2.org/). An individual with CF is commonly diagnosed through newborn screening programs, frequently by having elevated immunoreactive trypsinogen (IRT) and detection of bi-allelic pathogenic *CFTR* variants. To identify *CFTR* variants, many newborn screening programs use a panel that screens 23 to 40 variants that most commonly cause CF in Caucasian and Ashkenazi Jewish populations [5]. However the majority of pathogenic variants screened by defined panels do not represent the full spectrum of disease-causing variants in non-Caucasian populations, which can lead to delayed diagnosis and poorer health outcomes for individuals affected by CF in underrepresented ethnic groups such as Asians, Hispanics, and Africans [3].

Development of pan-ethnic CF panels that target the most common pathogenic variants across multiple ethnicities is one way to address the current Caucasian bias in molecular panels recommended for diagnosing CF [6]. However, in populations where large genomic datasets containing allele frequencies of the most common pathogenic *CFTR* variants are not currently available, such as for Indians, Indigenous peoples, or other ethnic minorities, development of pan-ethnic CF panels is challenging. Implementation of full gene sequencing of *CFTR* in screening programs, or as a diagnostic tool for symptomatic individuals, may be a better solution for having an unbiased method of identifying CF regardless of ethnic background. With the advent of more affordable and increasingly automated next-generation DNA sequencing (NGS) workflows and bioinformatics, NGS may provide scalable sequencing of *CFTR* and other genes in a cost-effective manner for screening programs and diagnostic laboratories to assist a diagnosis of CF.

Here we demonstrate how the problem of a “diagnostic odyssey” or missed diagnosis caused by the low sensitivity of CF mutation panels for non-Caucasians can be avoided by using NGS to sequence the entire *CFTR* gene as a second-tier newborn screening test. We report how full gene sequencing with NGS after a borderline IRT result removes the need for a multi-tiered strategy using targeted panels and allows a streamlined and racially equitable approach of identifying CF in an Indian patient carrying a rare pathogenic variant.

## Case presentation

The patient was a 4-month-old female born to non-consanguineous parents from a rural region in the state of Andhra Pradesh in south-east India. At 5 weeks of age the patient underwent newborn screening and was borderline for CF, with IRT levels of 88.5 μg/L with the normal range for newborn babies quoted is < 90 μg/L. Although the patient’s IRT level was slightly below the positive screen cut off value, second-tier testing was initiated due to a suspected family history of CF.

**Fig. 1:**
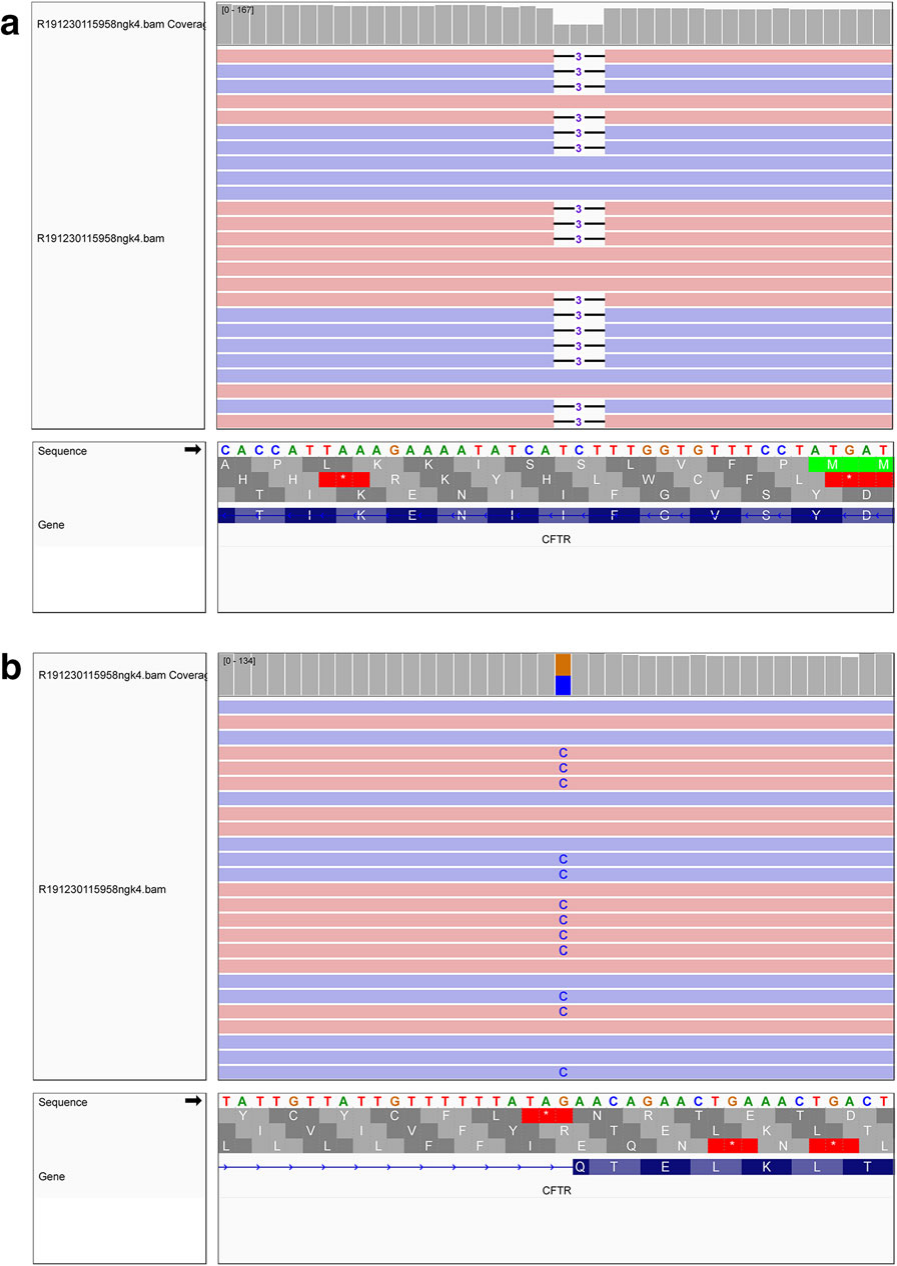
Identification of two pathogenic variants in *CFTR*. Next-generation DNA sequencing reads of the *CFTR* gene from the patient aligned to the reference sequence are visualized in the Integrative Genomics Viewer. Pink colored reads aligned to forward, and blue colored reads aligned to reverse DNA strands. **a** A pathogenic 3 base pair deletion is shown for the variant c.1521_1523delCTT. **b** A pathogenic single nucleotide variant c.870-1G > C is shown by the blue Cs.

Second-tier confirmatory genetic testing for CF by full gene sequencing using NGS as previously described [7], was used as part of a pilot study to investigate clinical utility of NGS in a newborn screening program. The NGS panel includes full sequencing of *CFTR* and the intronic regions flanking all exons. DNA variants were identified and classified according to American College of Medical Genetics and Genomics guidelines [8]. We found the patient was heterozygous for the NM_000492.3:c.1521_1523delCTT, p.(Phe508del), and heterozygous for the NM_000492.3:c.870-1G > C pathogenic variants in the *CFTR* gene (Fig. 1). The identification of two pathogenic variants supported the diagnosis of CF in the patient.

The c.1521_1523delCTT variant (also known as F508del) is the most common CF causing variant globally [9], and is included in all targeted CF panels. In contrast the c.870-1G > C splice-site variant is absent from gnomAD, ExAC and other population allele frequency databases, and is not included in targeted CF panels or currently listed in disease-specific variant databases such as CFTR2. The child’s mother was confirmed to be a carrier of the c.1521_1523delCTT variant, while the father did not consent to genotyping.

Due to financial constraints of the family and combined with lack of access to a public-funded dedicated CF Clinic, no specific treatment for CF was given and the child was discharged. The child presented to the hospital 6 months later and was pronounced dead on arrival. The baby was reported by the parents to have failure to thrive and had lower respiratory tract symptoms from 4 months of age. Two doses of oral amoxycillin by a local healthcare provider had been prescribed prior to death. The likely cause of death was from lower respiratory tract infection and respiratory failure. The exact cause of death is unknown because an autopsy was not performed, although it is assumed to be CF-related. An older sibling also had failure to thrive and died of a respiratory illness at the age of 1 year, and is thought to have had CF. No other information about the sibling is available.

## Discussion and conclusions

Second-tier testing by molecular CF panels restricted to targeting selected variants can lead to missed diagnosis and harmful “diagnostic odysseys” in individuals, especially non-Caucasians who are likely to have atypical pathogenic variants that will be missed by widely used CF panels [4, 10–14]. Our case study highlights how NGS can be used as a second-tier test to identify common and rare variants that cause CF to provide a racially equitable diagnosis pathway for a non-Caucasian. The rare variant identified in our patient is to our knowledge the first time to be reported in an Indian, although it has been observed in one Chinese family previously [15]. Further investigations will reveal if the variant is a more frequent cause of CF in the Indian and Chinese populations.

We were unable to gain consent for further diagnostic tests such as a sweat test, or to investigate pancreatic insufficiency in our patient. In the previously reported Chinese patient with genotype of c.3196C > T (p.R1066C) and c.870-1G > C, pancreatic insufficiency was indicated by steatorrhea and a positive faecal stool fat test [15]. Together with the CFTR2 database (last accessed November 24, 2020) listing p.R1066C as causing pancreatic insufficiency when combined with another variant that causes pancreatic insufficiency, there is evidence that c.870-1G > C also contributes to pancreatic insufficiency.

In our case, due to socioeconomic factors associated with the family living in a rural region of India, despite the rapid diagnosis of CF enabled by gene-sequencing the child died at 6 months. Although the parents had peace of mind from knowing the likely underlying cause of death, more support for CF patients is needed in India to improve clinical outcomes. It is anticipated that full-gene sequencing for CF diagnosis in countries where CF Clinics are accessible and other socioeconomic factors are more favorable would lead to a better outcome for affected individuals.

The rare variant we detected by NGS would have been missed by all targeted panels currently in use by most screening programs. For countries such as India, where the spectrum of pathogenic *CFTR* variants remain ill-defined, use of recommended targeted panels biased towards Caucasians will continue to delay diagnosis and cause harm to CF patients. Furthermore, by prioritizing targeted panels over full gene sequencing in racially diverse countries such as Australia, USA, and England, the inequality of healthcare for non-Caucasian CF patients is perpetuated and should be addressed. In the meantime, CF programs using targeted panels can mitigate the risk of racial bias through design of their confirmatory testing protocol, for example, by ensuring that when a patient’s IRT levels are high and no pathogenic variant is found by a panel, the patient is referred for further diagnostic tests.

Inclusion of full gene sequencing by NGS for CF into a newborn screening program may not be immediately appropriate for all programs and needs to be considered in the context of each programs wider health system. In some countries where CF treatment is scarce or unaffordable for example, resources may be better focused on accessibility to treatment, rather than increasing rates of diagnosis. Using NGS may be better suited to programs in countries with a large non-Caucasian population and where CF Clinics are readily accessible to improve the equity of current testing regimes.

NGS can detect single nucleotide variants, small insertions and deletions, mixed variants, large insertions and deletions, plus genotyping of the *CFTR* polymorphic TG-polyT tracts in one assay [16], making it an attractive method for second-tier newborn screening. However, use of full gene sequencing as a second-tier test is not currently used by most screening programs for a number of factors. One drawback of full gene sequencing is finding Variants of Unknown Significance (VUS) which causes clinical uncertainties [17]. In our laboratory which screens over 40 autosomal recessive diseases (including CF) as a first- or second-tier genomic newborn screening test, VUS that potentially cause clinical uncertainty is less than 0.01% of all cases. VUS do not cause significant clinical problems in our genomic screening test as the small number of ambiguous cases can be resolved by additional confirmatory testing through biochemical or other methods. We have also found the rates of homozygous VUS are very low in our genes of interest, which makes VUS less of a problem when screening for autosomal recessive conditions. The other scenario where VUS might cause clinical dilemmas is finding a carrier that also has a VUS in the same gene, but further confirmatory testing may help provide clinical certainty for some conditions. The uncertainties caused by VUS is similar to the challenges faced with setting cut-off values for biochemical assays that are commonly used as first tier screening tests. Equivocal results from non-genomic tests are widely tolerated in newborn screening for CF and other diseases [18, 19]. Therefore, the existence of VUS should not by itself preclude gene sequencing as a valid screening method. Appropriate pre- and post-test genetic counselling are nonetheless important considerations when considering implementation of NGS in newborn screening to minimize potential psychological harm from uncertain NGS results.

Functional assays can provide data to re-classify VUS and minimize their impact on sequencing results [20]. A comprehensive mutation screen across the entire *CFTR* gene using a novel and scalable functional assay could provide one mechanism to reduce the rates of VUS. Until VUS can be confidently classified as deleterious or tolerated, the benefits of being able to identify all known, and potentially new, CF-causing variants in one assay and equally for all individuals must be weighed up against the potential impact of identifying VUS.

Without second-tier full gene sequencing, there would likely have been a significantly delayed or missed diagnosis of CF in our Indian patient, unlike in a Caucasian patient whose pathogenic variants would in most cases have been identified by a targeted panel and who would have received a faster diagnosis. We show full gene sequencing of *CFTR* by NGS is a useful second-tier test for diagnosing CF. Until full gene sequencing is more widely used for first- or second-tier CF screening in newborn screening programs, non-Caucasian CF patients will continue to suffer from a higher level of harm through delayed diagnosis and repeated testing [4, 21].

## Data Availability

The datasets used and/or analyzed during the current study are available from the corresponding author on reasonable request.

## Abbreviations

CF: Cystic Fibrosis
CFTR: Cystic fibrosis transmembrane conductance regulator
NGS: Next-generation DNA sequencing
VUS: Variants of Unknown Significance
IRT: Immunoreactive trypsinogen
